# Anti-sortilin1 Antibody Up-Regulates Progranulin *via* Sortilin1 Down-Regulation

**DOI:** 10.3389/fnins.2020.586107

**Published:** 2020-12-15

**Authors:** Shuuichi Miyakawa, Hiroyuki Sakuma, Dnyaneshwar Warude, Satomi Asanuma, Naoto Arimura, Tomoki Yoshihara, Daniel Tavares, Akito Hata, Koh Ida, Yuri Hori, Yuumi Okuzono, Syunsuke Yamamoto, Koichi Iida, Hisao Shimizu, Shinichi Kondo, Shuji Sato

**Affiliations:** ^1^Immunology Unit, Research, Takeda Pharmaceutical Company Limited, Fujisawa, Japan; ^2^Global Biologics Research, Takeda Pharmaceutical Company Limited, Cambridge, MA, United States; ^3^Drug Metabolism and Pharmacokinetics Research Laboratories, Research, Takeda Pharmaceutical Company Limited, Fujisawa, Japan

**Keywords:** sortilin1 (SORT1), progranulin (PGRN), granulin (GRN), monoclonal antibody, frontotemporal dementia

## Abstract

Progranulin (PGRN) haploinsufficiency associated with loss-of-function mutations in the granulin gene causes frontotemporal dementia (FTD). This suggests that increasing PGRN levels could have promising therapeutic implications for patients carrying *GRN* mutations. In this study, we explored the therapeutic potential of sortilin1 (SORT1), a clearance receptor of PGRN, by generating and characterizing monoclonal antibodies against SORT1. Anti-SORT1 monoclonal antibodies were generated by immunizing *Sort1* knockout mice with SORT1 protein. The antibodies were classified into 7 epitope bins based on their competitive binding to the SORT1 protein and further defined by epitope bin-dependent characteristics, including SORT1-PGRN blocking, SORT1 down-regulation, and binding to human and mouse SORT1. We identified a positive correlation between PGRN up-regulation and SORT1 down-regulation. Furthermore, we also characterized K1-67 antibody via SORT1 down-regulation and binding to mouse SORT1 *in vivo* and confirmed that K1-67 significantly up-regulated PGRN levels in plasma and brain interstitial fluid of mice. These data indicate that SORT1 down-regulation is a key mechanism in increasing PGRN levels via anti-SORT1 antibodies and suggest that SORT1 is a potential target to correct PGRN reduction, such as that in patients with FTD caused by *GRN* mutation.

## Introduction

Frontotemporal dementia (FTD) is a neurodegenerative disease characterized by the selective and progressive degeneration of the frontotemporal lobe. The disease is associated with progressive dementia, behavioral changes, and altered sociability and requires extensive medical care. Currently, the only available remedies for FTD involve symptomatic treatment that does not slow disease progression. Genome-wide association studies and mutation analysis of FTD patients have identified specific genes as risk factors for inherited FTD, including *GRN*, *C9orf72*, *MAPT*, *TMEM106B*, and *CST3* (Benussi et al., 2010; Pottier et al., 2016). *GRN* mutations are responsible for 5–20% of familial FTD cases and 1–12% of sporadic cases (Rademakers et al., 2012). Most *GRN* mutations result in a reduction in its protein product, progranulin (PGRN), via non-sense-mediated mRNA decay. This leads to PGRN haploinsufficiency (Ward and Miller, 2011). Patients with *GRN* mutations have reduced PGRN levels in their plasma, serum, or CSF: only 30–50% of normal levels (Ghidoni et al., 2008; Mukherjee et al., 2008; Van Damme et al., 2008; Finch et al., 2009; Sleegers et al., 2009). These findings suggest that boosting PGRN levels could be a promising therapy for FTD treatment. A recent preclinical study has supported this notion by demonstrating that adeno-associated virus-driven expression of PGRN in the medial prefrontal cortex rescued social dominance deficits in a FTD model of *Grn* hetero-KO mice (Arrant et al., 2017). Drug discovery research has also investigated PGRN-boosting therapies *in vitro* by targeting epigenetic factors and transcription factors (Capell et al., 2011; Cenik et al., 2011; Holler et al., 2016; Elia et al., 2020). However, these approaches have not been tested *in vivo*.

PGRN is a widely distributed pleiotropic protein that consists of seven and half cysteine-rich repeats (Mendsaikhan et al., 2019). In the brain, PGRN is secreted from microglia and acts as a neurotrophic factor, regulating a diverse range of cellular functions including cell proliferation, neuron survival, cell migration, neurite extension, lysosomal function, and anti-inflammatory responses (Toh et al., 2011; Kao et al., 2017). Sortilin 1 (SORT1), a type I transmembrane glycoprotein, is a clearance receptor of PGRN that acts by facilitating PGRN internalization (Hu et al., 2010). SORT1 polymorphisms have been linked to PGRN levels in serum, as well as altered susceptibility to FTD and Alzheimer’s disease (McMillan et al., 2014; Andersson et al., 2016; Philtjens et al., 2018; Tönjes et al., 2018), suggesting a key role of SORT1 in the regulation of PGRN levels. This notion is also supported by the observations that (1) *Sort* KO raises *in vivo* PGRN levels by 2.5- to 5-fold and (2) *Sort1* ablation reverses the decrease in PGRN levels observed in *Grn* hetero-KO mice (Hu et al., 2010). In fact, the biotech company Alector is testing an anti-SORT1 antibody in phase 3 clinical trials for the treatment of FTD, and is recruiting patients to evaluate the efficacy of the anti-SORT1 antibody (ClinicalTrials.gov, 2020).

In this study, we generated a variety of anti-SORT1 monoclonal antibodies (mAbs) to validate this hypothesis and establish their utility as potential therapeutics for FTD attributed to *GRN* mutations. Here, we describe the characteristics of these mAbs and discuss how they influence PGRN levels.

## Results

### Generation of Anti-SORT1 mAbs

To assess whether reducing SORT1 function can up-regulate extracellular PGRN levels, we generated and characterized anti-SORT1 mAbs, that were cross-reactive to human and mouse SORT1. To do this, we first immunized WT mice with human SORT1 recombinant protein but unfortunately this approach produced anti-SORT1 antibodies that bound to human but not to mouse SORT1, perhaps because of immunotolerance to self-antigen. In an attempt to overcome this failure, we next decided to use *Sort1* KO mice, naïve to mouse SORT1, and immunized them with human SORT1 protein (first to fifth immunization) and mouse SORT1 protein (sixth to tenth immunization) sequentially. To effectively obtain anti-SORT1 mAbs, an anti-mouse CD25 mAb was intraperitoneally injected into 4 *Sort1* KO mice 2 days before the first immunization. This tactic was utilized based on a previous finding that CD25-positive T cell depletion enhances antibody response (Ndure and Flanagan, 2014). The immunized mice were bled after the fifth and ninth immunizations to establish antibody titers against SORT1 by FCM using SORT1 expressing cells. We sacrificed the mice and screened hybridomas derived from lymphocytes from popliteal lymph nodes to identify anti-SORT antibody expressors. The assay identified 29 hybridoma clones producing antibodies which cross-reacted to human and mouse SORT1 from 2,300 wells of hybridomas. The 29 anti-SORT1 mAbs were then purified from hybridoma supernatants for further characterization.

### Characterization of Anti-SORT1 mAbs

To characterize the anti-SORT1 mAbs, we performed multiple *in vitro* assays including binding ELISA, epitope binning, PGRN up-regulation assay using human and mouse cells, SORT1 down-regulation assay, and PGRN-SORT1 blocking assay. First, we confirmed the binding of mAbs to human and mouse SORT1 by ELISA and found that each anti-SORT1 mAb showed different binding characteristics toward human and mouse SORT1. These results indicate that our human and mouse cross-reactive anti-SORT1 mAbs have a wide range of cross-reactivity (Table 1).

**TABLE 1:** Summary of anti-SORT1 mAb characteristics.

				Human U251 cell			Mouse primary neuron		
Clone	Epitope bin^a^	hSORT1-His ELISA^b^ (OD @450 nm)	hPGRN-hSORT1 binding^c^ (% inhibition)	SORT1 down-regulation^d^ (% control)	PGRN up-regulation^e^ (fold-change)	mSORT1-His ELISA^f^ (OD @450 nm)	SORT1 down-regulation^g^ (% control)	PGRN up-regulation^h^ (fold-change)	Function^i^ (PGRN competition or SORT1 down-regulation)
K1-19	I	1.44	13.2	95.4***	2.40***	0.78	4.2	1.13*	SORT1 down-regulation
K1-32	I	1.48	22.7	95.7***	2.20***	0.83	15.7	1.14*	SORT1 down-regulation
K1-52	I	0.19	80.5	44.1***	1.10*	0.94	17.3	1.17*	
K1-65	I	0.95	14.2	85.9***	1.70**	0.53	19.6	1.19	
K1-70	I	0.31	17.7	4.3	1.00	0.46	11.7	1.09*	
K1-12	II	0.78	58.1	87.1***	1.80***	0.78	53.5**	2.32***	
K1-66	II	0.14	34.6	31.9***	1.00	0.19	16.6	1.40**	
K1-15	III	0.70	90.7	71.1***	2.00**	1.36	17.0*	2.49***	PGRN competition
K1-27	III	0.49	76.9	76.2***	1.90*	1.43	28.8*	1.47**	
K1-44	III	0.41	89.8	75.7***	1.96***	1.04	36.4**	1.83***	
K1-54	III	0.54	99.5	75.1***	1.90**	1.33	33.6*	1.74**	PGRN competition
K1-62	III	0.22	96.3	13.7	1.00	0.15	5.4	1.14*	PGRN competition
K1-68	III	0.38	86.1	80.1***	2.00**	1.23	40.5*	1.65**	
K1-11	IV	0.25	99.2	62.6***	1.90**	1.17	35.7*	1.83*	PGRN competition
K1-24	IV	0.16	27.1	63.8**	1.40**	0.23	46.7**	1.39**	
K1-08	V	1.83	41.6	88.2***	2.00***	1.57	66.4**	1.80***	
K1-09	V	1.97	47.2	84.7***	1.60**	1.76	67.2**	1.78**	
K1-13	V	1.91	31.9	95.0***	1.90**	1.86	53.1**	1.55**	SORT1 down-regulation
K1-40	V	2.01	14.5	94.1***	2.30***	1.90	65.5**	2.11***	SORT1 down-regulation
K1-47	V	0.32	22.1	7.4	0.90	0.43	9.8	1.06	
K1-48	V	1.62	-3.5	95.4***	1.80**	1.83	62.2**	2.29**	SORT1 down-regulation
K1-61	V	1.42	33.6	85.1***	1.60***	1.17	53.9**	1.70**	
K1-67	VI	1.00	63.0	95.2***	1.90**	0.28	67.7***	2.07***	SORT1 down-regulation
K1-71	VI	0.43	46.8	88.1***	1.90**	0.14	37.9***	1.28*	
K1-02	VII	0.07	86.6	31.7**	1.10	1.37	43.1**	2.01**	
K1-04	VII	0.37	95.0	58.9***	1.60**	1.57	31.4*	1.78**	PGRN competition
K1-05	VII	0.13	102.0	16.2**	1.00	1.37	36.4**	1.83***	PGRN competition
K1-06	VII	0.13	101.4	31.1**	1.10	1.62	40.2*	1.89**	PGRN competition
K1-07	VII	0.17	100.0	12.1**	1.10	1.44	42.4**	1.70**	PGRN competition

^a^Epitope bin was determined by competitive binding profiles of anti-SORT1 mAbs in sandwich ELISA. ^b,f^Binding activity of anti-SORT1 mAb is shown as absorbance at 450 nm determined using ELISA. ^c^PGRN-SORT1 interaction block by anti-SORT1 mAb was calculated as a % inhibition at 15 μg/mL (vs. PBS-treated control group). ^e,h^PGRN up-regulation by anti-SORT1 mAb was calculated as a fold-change (vs. PBS-treated control). * p < 0.05, ** p < 0.01, *** p < 0.001 vs. PBS-treated group by Student’s t-test.^d,g^SORT1 down-regulation is represented as SORT1 levels down-regulated from cell surface in response to anti-SORT1 mAb. * p < 0.05, ** p < 0.01, *** p < 0.001 vs. PBS-treated group by Student’s t-test.^i^The inhibition of the interaction between PGRN and SORT1 in more than 90% was classified as PGRN competition. The down-regulation of SORT1 in more than 90% was classified as SORT1 down-regulation.

**FIGURE 1:**
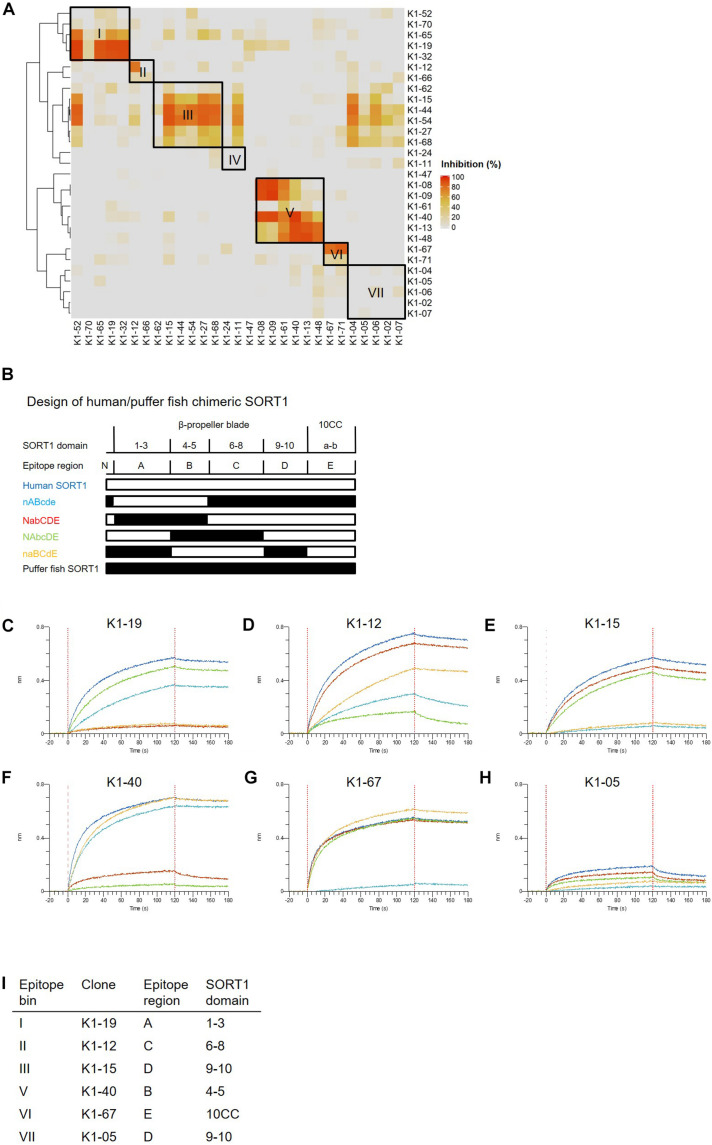
Binding profiles of anti-SORT1 mAbs. **(A)** Epitope binning of anti-SORT1 mAbs. The competitive binding was measured in a sandwich ELISA with 29 anti-SORT1 mAbs. Each column and row in the matrix represent an unlabeled and biotinylated anti-SORT1 mAb. The competitive binding of the 29 anti-SORT1 mAbs is shown as a heat map analyzed by Ward’s hierarchical clustering. The color scale from 0 to 100 shows the competitive binding of the 2 mAbs. **(B)** Design of chimeric proteins; nABcde (human SORT1 A, B and puffer fish SORT1 N-terminal C, D, E regions), NabCDE (human SORT1 N-terminal, C, D, E and puffer fish SORT1 A, B regions), NAbcDE (human SORT1 N-terminal, A, D, E and puffer fish SORT1 B, C regions), and naBCdE (human SORT1 B, C, E and puffer fish SORT1 N-terminal, A, D regions). The white bar indicates the SORT1 domain derived from human and the black bar indicates the SORT1 domain derived from puffer fish. **(C–H)** Epitope mapping of anti-SORT1 mAbs. Epitope mapping was analyzed by the binding pattern of anti-SORT1 mAb to human/puffer fish chimeric SORT1 proteins in BLI. The BLI analysis was performed with anti-SORT1 mAb K1-19 **(C)**, K1-12 **(D)**, K1-15 **(E)**, K1-40 **(F)**, K1-67 **(G)**, and K1-05 **(H)** as ligands and chimeric SORT1 protein as an analyte. The vertical axis indicates the BLI signal response (nm). The horizontal axis indicates the time after analyte loading. **(I)** Epitope mapping results of anti-SORT1 mAbs tested are shown in the table.

We then classified anti-SORT1 mAbs by epitope binning based on a competitive sandwich ELISA method. Epitope binning is widely used for clustering mAbs by the competitive binding pattern among mAbs. To perform epitope binning, anti-SORT1 mAb was immobilized in an ELISA plate, and then a complex of a different anti-SORT1 mAb (competitor mAb) and SORT1 protein was added to the plate. Wherever epitopes of the immobilized mAb and competitor mAb did not overlap, SORT1 protein can be captured by both the mAbs separately. However, when epitopes of immobilized mAb and competitor mAb overlap, immobilized mAb and competitor mAb competitively bind to SORT1, and the competitive binding can be detected as a reduced signal of competitor mAb (biotinylated). The competitive sandwich ELISA result was obtained as the 29 × 29 matrix of binding inhibition (%). After Ward’s hierarchical clustering, the 29 mAbs fell into 7 bins (Figure 1A).

We then determined the binding region of mAbs of each epitope bin in the following epitope mapping study. It has previously been reported that the extracellular domain of the SORT1 protein consists of three domains; a ten-bladed β-propeller and two 10CC domains (10CC-a and 10CC-b) (Quistgaard et al., 2014). Based on this information, we generated chimeric SORT1 proteins of human and a xenogenic species by domain-swapping. We renamed the domains for the purpose of convenience in this study. Since mature SORT1 is released by furin cleavage from its proform at amino acid residue 77 (Petersen et al., 1997), we used this information to design chimeric proteins : N-terminal region (Arg77–Arg109), A region (1–3 β-propeller blade), B region (4–5 β-propeller blade), C region (6–8 β-propeller blade), D region (9–10 β-propeller blade), and E region (10CC-a and 10CC-b).

For this purpose, we chose Takifugu rubripes (puffer fish) SORT1 protein (UniProt accession H2RV63), which shares 56.2% amino acid identity with human SORT1 (UniProt accession Q99523). We generated 4 chimeric proteins; nABcde (human SORT1 A, B and puffer fish SORT1 N-terminal, C, D, E regions), NabCDE (human SORT1 N-terminal, C, D, E and puffer fish SORT1 A, B regions), NAbcDE (human SORT1 N-terminal, A, D, E and puffer fish SORT1 B, C regions), and naBCdE (human SORT1 B, C, E and puffer fish SORT1 N-terminal, A, D regions) (Figure 1B). The binding of anti-SORT1 mAbs to the chimeric SORT1 proteins was detected by BLI. Anti-SORT1 mAb was captured by AMC biosensor, and then chimeric SORT1 proteins were applied as an analyte. We applied 6 anti-SORT1 mAbs (K1-19, K1-12, K1-15, K1-40, K1-67, and K1-05) as representatives of epitope bins I, II, III, V, VI, and VII, respectively, in this epitope mapping study. Due to the low binding of epitope bin IV mAbs to human SORT1 protein, we did not include epitope bin IV mAb in this epitope mapping study. Each anti-SORT1 mAb demonstrated a distinctive binding pattern. Epitope bin I mAb, K1-19 bound to nABcde and NAbcDE, as well as to human SORT1 (Figure 1C). Meanwhile, K1-19 failed to bind to NabCDE and naBCdE (Figure 1C). These results indicate that K1-19 binds to the A region, which is shared among chimeric proteins. Similarly, other anti-SORT1 mAbs were analyzed and their epitopes were mapped by their chimeric protein binding patterns. The epitope of K1-12, K1-15, K1-40, K1-67, or K1-05 were identified as C, D, B, E, or D region, respectively (Figures 1C–I).

Recent discoveries have demonstrated that SORT1 is a clearance receptor for PGRN, promoting PGRN endocytosis and thereby determining plasma PGRN levels (Hu et al., 2010). This led us to investigate whether anti-SORT1 mAbs increased the PGRN concentration in the extracellular environment by blocking SORT1 function. We treated U251 human glioblastoma cells, which have the inherent capacity to release PGRN, with the anti-SORT1 antibody (Li et al., 2017). In the U251 PGRN assay, 19 out of 29 mAbs tested showed more than a 1.5-fold increase of extracellular PGRN compared to the control group (Table 1). However, a few among the 29 mAbs had no impact on extracellular PGRN levels. We then assessed whether mAbs were able to demonstrate a similar effect in mouse by using primary cortical neurons. Among the 29 mAbs, 18 mAbs raised extracellular mouse PGRN by more than 1.5 times compared to that in the control group. Intriguingly, 14 out of the 18 mAbs were among the 19 mAbs increasing human PGRN levels in the U251 assay. The exceptions were clones K1-02, K1-05, K1-06, and K1-07 demonstrating a mouse-specific PGRN increase, likely due to their relatively weak human versus mouse SORT1 binding ability as per the binding ELISA data (Table 1). We then assessed if there was a correlation between SORT1-binding and PGRN up-regulation between species. ELISA binding activity of anti-SORT1 mAb was found to be linked to the extent of PGRN up-regulation (Pearson correlation coefficient: human species, *r* = 0.63, *p* = 2.8 × 10^–4^; mouse species, *r* = 0.56, *p* = 1.4 × 10^–3^). These results suggested that the ability of an anti-SORT1 mAb to up-regulate PGRN was dependent on its binding affinity to SORT1. ELISA binding activities of anti-SORT1 mAbs to human and mouse SORT1 also showed a moderate correlation (*r* = 0.43, *p* = 0.02). However, the correlation between human and mouse PGRN up-regulation was not significant (*r* = 0.27, *p* = 0.16).

To investigate the molecular mechanisms by which anti-SORT1 mAbs increased PGRN levels, we conducted two assays based on the following assumptions: anti-SORT1 mAb would inhibit PGRN binding to SORT1 and/or would decrease membrane-bound SORT1 levels by down-regulating SORT1 protein. First, we performed SORT1-PGRN interaction analysis in which biotinylated PGRN remained on cells transiently over-expressing human SORT1 was detected in the presence of anti-SORT1 mAb. Fifteen out of 29 mAbs were able to block the SORT1-PGRN interaction by more than 50% at 15 μg/mL. The inhibitory activity of mAb against the SORT1-PGRN interaction showed no significant correlation with PGRN up-regulation (human species, *r* = −0.23, *p* = 0.23; mouse species, *r* = 0.28, *p* = 0.14). We then tested how anti-SORT1 mAb would affect membrane-bound SORT1 levels. In our study, 29 mAbs down-regulated SORT1 protein levels to different extents (Table 1). This effect was found to have a moderate correlation between human and mouse (*r* = 0.47, *p* = 0.01). Intriguingly, the SORT1 down-regulation was strongly correlated with PGRN up-regulation in human species (*r* = 0.9, *p* = 2.7 × 10^–11^) and moderately in mouse species (*r* = 0.63, *p* = 1.7 × 10^–4^). These findings indicate that SORT1 down-regulation triggered by the anti-SORT1 mAbs contributed to the up-regulation of PGRN.

These characterization assays define epitope bin-dependent activities of the anti-SORT1 antibodies. Some antibodies, such as K1-19 and K1-32, up-regulated PGRN levels only in human cells. Both antibodies belonged to epitope bin I and only showed strong binding to the human SORT1 molecule, suggesting that the epitope recognized by bin I mAbs is a human-specific sequence in SORT1. K1-12 in epitope bin II showed a moderate PGRN up-regulation in both human and mouse cells. On the other hand, epitope bin VII mAbs, such as K1-05, showed moderate PGRN up-regulation in mouse neurons but almost no activity in human cells. K1-67 from epitope bin VI showed a strong PGRN up-regulation in both human and mouse cells with high SORT1 down-regulation. The anti-SORT1 mAbs derived from bin III and bin VII blocked the SORT1-PGRN interaction. Both bin III and bin VII mAbs interacted with the D region of the SORT1 molecule. The bin III mAbs did not show strong SORT1 down-regulation, while bin VII mAbs did. These results clearly demonstrate the epitope bin defining characteristics of the antibodies discovered in this study.

Through this profiling, we found that SORT1 down-regulation was strongly correlated with PGRN up-regulation. The epitope bin VI mAb, K1-67 was selected for further evaluation due to its human and mouse cross-reactivity, high SORT1 down-regulation, potent PGRN up-regulation, and no PGRN-SORT1 interaction blocking activity.

### Characterization of Anti-SORT1 mAb K1-67

**FIGURE 2:**
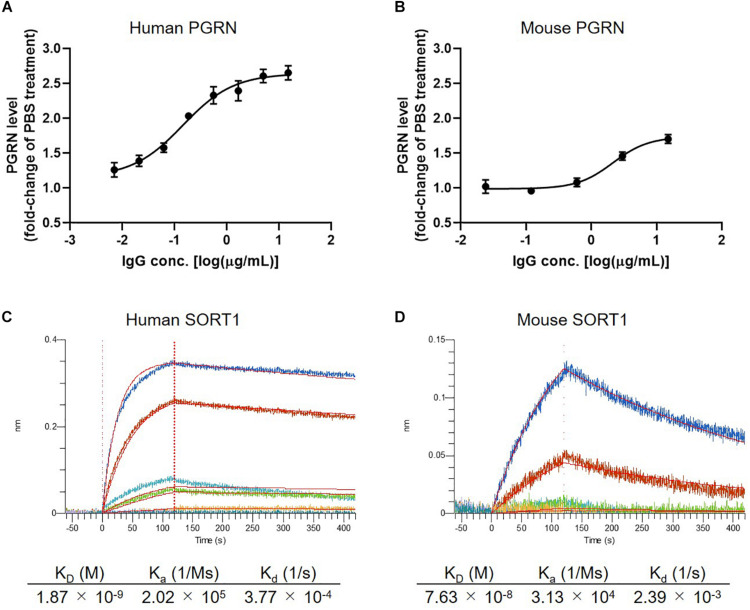
Detailed characterization of anti-SORT1 mAb K1-67. **(A)** PGRN up-regulation in response to K1-67 in U251 human glioblastoma cells. Cells were treated with various concentrations of K1-67 for 72 h. Human PGRN in U251 culture supernatant was determined by ELISA and is shown as a fold-change relative to PBS-treated cells. Data are mean ± SEM from 3 independent experiments. **(B)** PGRN up-regulation in response to K1-67 in mouse primary neurons. Cells were treated with various concentrations of K1-67 for 7 days. Mouse PGRN in mouse primary neuron culture supernatant was determined by ELISA and is shown as a fold-change relative to PBS-treated cells. Data are mean ± SEM from 3 independent experiments. **(C,D)** Affinity of K1-67 to SORT1. BLI was used to determine the affinity of K1-67 toward human and mouse SORT1 with K1-67 as a ligand and with SORT1-His protein as an analyte. The vertical axis indicates the BLI signal response (nm), and the horizontal line indicates the time after analyte loading. Kinetic parameters were analyzed using a 1:1 Langmuir fitting model. Association (*K*_a_) and dissociation (*K*_d_) constants were calculated and used to determine the *K*_D_ value (*K*_d_/*K*_a_).

In the first set of experiments we tested the concentration-dependency of PGRN up-regulation by K1-67 using U251 cells and mouse primary neurons. K1-67 up-regulated PGRN levels in both U251 cells and mouse primary neurons in a concentration-dependent manner with EC_50_ values of 0.14 and 2.14 μg/mL, respectively (Figures 2A,B). We also determined the affinity of K1-67 to SORT1. The *K*_D_ values of K1-67 to human and mouse SORT1 were 1.87 × 10^–9^ M and 7.63 × 10^–8^ M, respectively, according to an analysis using a Langmuir fitting model (Figures 2C,D). The *K*_a_ of the antibody was 2.02 × 10^5^ (1/M/s) and the *K*_d_ was 3.77 × 10^–4^ (1/s) toward human SORT1. Meanwhile, the *K*_a_ of K1-67 was 3.13 × 10^4^ (1/M/s) and the *K*_d_ was 2.39 × 10^–3^ (1/s) toward mouse SORT1.

### Plasma and CSF PGRN Up-Regulation in K1-67-Treated Mice

**FIGURE 3:**
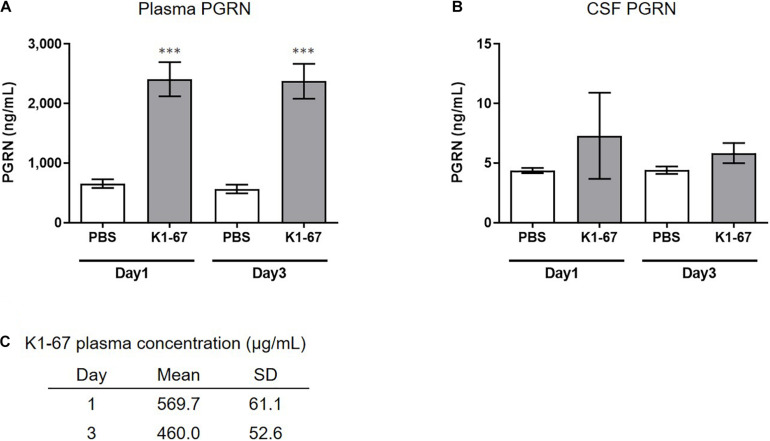
Up-regulation of plasma or CSF PGRN levels in K1-67-treated mice. **(A,B)** Plasma or CSF PGRN levels in response to K1-67. Mouse plasma or CSF was collected 1- or 3-days after the treatment of K1-67 at 100 mg/kg, i.v., and subjected to ELISA. Pooled CSF from 4 mouse was used due to the small volume of CSF from individual mouse. The vertical axis indicates PGRN concentration. Data are mean ± SD from 12 or 3–4 independent experiments for plasma and CSF, respectively. ^∗∗∗^*p* < 0.001 vs. PBS-treated group by Aspin–Welch *t*-test. **(C)** K1-67 concentration in plasma. Mouse plasma was collected 1 or 3 days after the treatment of K1-67 at 100 mg/kg, i.v., and subjected to liquid chromatography-mass spectrometry. Data are mean ± SD from 3 independent experiments.

We next tested if down-regulation of SORT1 by anti-SORT1 mAb was able to induce PGRN up-regulation *in vivo*. It is well known that the BBB prevents the delivery of large molecules, such as proteins or antibodies, to the brain (Pardridge, 2003). To detect PGRN up-regulation, as shown in the mouse primary neuron assay (Figure 2B), we speculated that K1-67 concentration would need to be more than 1 μg/mL, which would be achieved with a plasma concentration of 200 μg/mL to 1 mg/mL, assuming that the concentration of antibodies in the brain after peripheral treatment is as low as 0.1–0.5% of the concentration in the blood (Shin et al., 1995; Pardridge, 2005; Boado et al., 2013). One or 3 days after a single intravenous administration of K1-67 at 100 mg/kg, plasma concentration of K1-67 reached 570 μg/mL or 460 μg/mL, respectively, and there was a trend for an increase in PGRN level in CSF (Figures 3A–C).

### Interstitial Fluid (ISF) PGRN Up-Regulation in K1-67-Treated Mice

**FIGURE 4:**
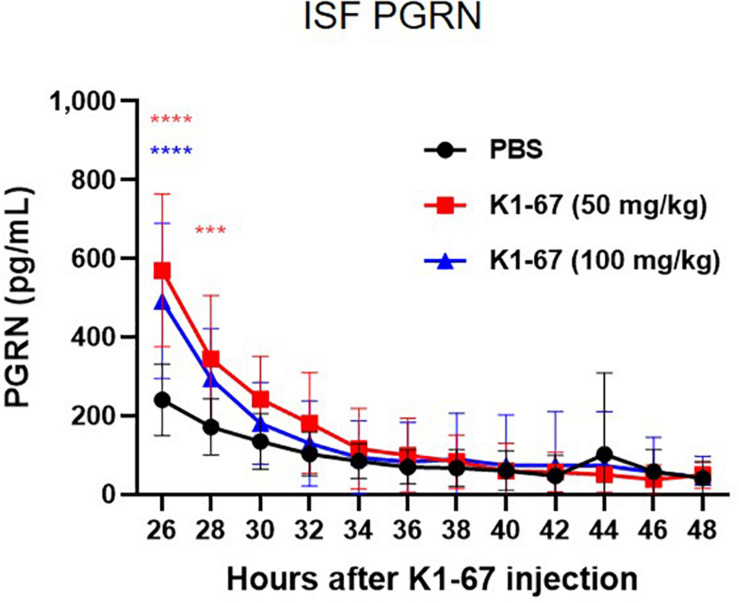
ISF PGRN levels in response to K1-67. Mouse ISF was collected from a probe in hippocampal microdialysis over 24 h after the treatment of K1-67 at 50 mg/kg or 100 mg/kg, i.v. PGRN levels were determined by ELISA. The vertical axis indicates PGRN concentration. The horizontal axis indicates the hours after K1-67 injection. Data are mean ± SD from 10 independent experiments. ***p < 0.001, ****p < 0.0001 vs. PBS by Two-way RM ANOVA analysis with Sidak’s multiple comparisons.

In mouse brain, CSF has been reported to be produced at a rate of 350 nL/min, which means the total CSF volume of 40 μl turns over 12–13 times per day (Johanson et al., 2008). This led us to think that the rapid CSF turnover may have influenced the effect of K1-67 on CSF levels and diminished the up-regulation of CSF PGRN levels. Therefore, we considered measuring PGRN levels in the ISF, the turnover of which is reportedly lower than that of CSF (Abbott, 2004). To do this we used a microdialysis method, in which ISF could be continuously collected from a probe implanted in the hippocampus and efflux of PGRN could be minimized. We administered K1-67 at two doses of 100 mg/kg, which we speculated was high enough to achieve PGRN up-regulation in the brain, and half of the dose, 50 mg/kg into mice 24 h prior to the first collection of microdialysates. As shown in Figure 4, we observed a continuous decline of ISF PGRN levels over time in the PBS-treated group, which we speculate was due to probe membrane clogging by glial cells (unpublished observation). Meanwhile, we found significant up-regulation of ISF PGRN levels by K1-67 at after 2 and 4 h of microdialysate collection (26 and 28 h after K1-67 treatment, respectively). This result clearly indicated that anti-SORT1 mAb K1-67, boosted ISF PGRN levels in the brain.

## Discussion

### Expected Benefits of Anti-SORT1 mAb Compared With Reported PGRN Up-Regulation

In this study, we demonstrated that SORT1 down-regulation mediated by anti-SORT1 mAb, rather than SORT1-PGRN binding inhibition, led to the up-regulation of PGRN levels. This notion may be useful in exploring SORT1-mediated PGRN up-regulators, as SORT1 is a direct clearance receptor of PGRN. Other compounds, excluding anti-SORT1 mAbs, have been identified to be effective in increasing PGRN levels in cell lines derived from *GRN* mutation carriers (Capell et al., 2011; Cenik et al., 2011; Holler et al., 2016; Elia et al., 2020). Inhibitors of histone deacetylases (HDACs) was found to increase PGRN gene and protein expression through unknown mechanisms (Cenik et al., 2011). V-ATPase inhibitors enhanced PGRN expression or significantly elevated PGRN secretion via a translational mechanism independent of lysosomal degradation, autophagy, or endocytosis (Capell et al., 2011). Pharmacological inhibition of FOXO1, TRAP1/HSP90L, JMJD6, or ELK3 increased PGRN levels in neurons, inhibiting the activity of their gene products (Elia et al., 2020). Trehalose transcriptionally up-regulates PGRN expression in human and mouse models of GRN haploinsufficiency, and it rescues PGRN deficiency in human fibroblasts and neurons derived from induced pluripotent stem cells generated from *GRN* mutation carriers (Holler et al., 2016). While these drug inhibitors that target genetic modifications of PGRN expression may have potential in treating FTD by up-regulating PGRN, they may produce off-target actions. HDAC inhibitors decrease cell viability at the concentrations that enhance PGRN levels (Cenik et al., 2011). Additionally, none of the abovementioned molecules have been reported to have a PGRN-specific mechanism, unlike SORT1 which is a PGRN clearance receptor. Instead, they act as global regulators of cell functions, such as transcriptional or mitochondrial functions. Previous reports have identified SORT1 binding compounds (Andersen et al., 2014; Schrøder et al., 2014) but their effect on PGRN up-regulation is unknown.

### SORT1 Down-Regulation Is a Key Function to PGRN Up-Regulation

Our analyses revealed that SORT1 down-regulation activity is essential for up-regulation of PGRN by anti-SORT1 mAb. To date, several therapeutic antibodies have been reported to down-regulate the expression of target proteins (Fan et al., 1994; Chiavenna et al., 2017). The molecular mechanism of antibodies in down-regulation is not fully understood, but it is thought that the binding of the antibody leads to removal of the target protein from the cell surface and alteration of the intracellular fate, resulting in accelerated degradation of the target protein. Considering that SORT1 plays roles in the trafficking of multiple proteins between the cell surface and lysosome, we assume that anti-SORT1 mAb efficiently uses this property of SORT1 as a protein shuttle to down-regulate itself (Canuel et al., 2008; Hermey, 2009; Musunuru et al., 2010).

### Region D in SORT1 Is Required for PGRN-SORT1 Interaction

We discovered several types of anti-SORT1 mAbs which blocked PGRN-SORT1 interaction and/or down-regulated SORT1 down-regulation. A previous report has suggested that PGRN binds to the beta-propeller region of SORT1 through its C-terminal tail (Zheng et al., 2011). Furthermore, another report using HDX-MS technology indicated that PGRN binds to two separate surfaces opposite each other in the cavity of the beta-propeller region of SORT1 and induces conformational dynamics of SORT1 upon binding (Trabjerg et al., 2019). In our study, the mAbs in epitope bins III and VII blocked PGRN-SORT1 interaction and the epitope region of these mAbs was identified as the D region. The beta-propeller region of SORT1 includes the other regions of A, B, and C, suggesting that the D region in SORT1 is the largest contributor to the interaction with PGRN.

### Potential Therapeutic Application in the Treatment of FTD

In several studies, plasma and CSF PGRN levels were measured in *GRN* mutant carriers and were found to be significantly lower in FTD patients with *GRN* mutations than in non-mutation carriers, and plasma PGRN was correlated with CSF PGRN in *GRN* mutation carriers (Nicholson et al., 2014). Similarly, low levels of PGRN were observed even in asymptomatic *GRN* mutation carriers (Finch et al., 2009; Galimberti et al., 2018). PGRN levels in *GRN* mutation carriers are 2–3 times lower than in non-mutation carriers and inducing a two—threefold increase in PGRN levels could have a therapeutic potential for diseases caused by *GRN* mutation in a haplosufficient manner. In our study, after administration of the anti-SORT1 mAb K1-67, an approximate twofold increase in PGRN level was observed in WT mouse brain ISF, demonstrating the potential of anti-SORT1 mAb for increasing PGRN levels. Recently, important roles of PGRN in the lysosome have been discovered and reviewed elsewhere (Paushter et al., 2018). Rare homozygous mutations in *GRN* were shown to cause a juvenile onset lysosomal storage neurodegenerative disorder called neuronal ceroid lipofuscinosis (NCL) (Smith et al., 2012). In agreement with this study, *Grn* KO mice showed lysosomal dysfunction, including lipofuscin deposits and defects in lysosomal turnover (Ahmed et al., 2010; Wils et al., 2012); moreover, FTD patients with *GRN* mutations exhibit lipofuscin deposits in their cortex and retina and fibroblasts derived from these patients have decreased lysosomal protease activity (Ward et al., 2017). Additionally, PGRN is proteolytically processed into GRN peptides in the lysosome (Holler et al., 2017; Zhou et al., 2017) and both PGRN and GRN levels were found to be reduced in brain lysates from FTD patients with *GRN* mutation (Holler et al., 2017). The authors also revealed that *SORT1* KO HAP1 cells presented increased levels of PGRN and decreased levels of GRN peptides, suggesting that SORT1 plays a role in transporting intracellular PGRN to the lysosome. At the same time, they found that the genetic deletion of *SORT1* did not completely eliminate the production of GRN, which may be controlled by other pathways such as the prosaposin pathway. The anti-SORT1 mAb effect on lysosomal PGRN and GRN peptides may not be the same as that seen in *SORT1* KO cells. However, further studies are needed to validate the effect of anti-SORT1 mAb on intracellular PGRN and GRN peptides and specifically, to demonstrate that anti-SORT1 approach does not reduce the lysosomal pool.

### Effect of SORT1 Blocking on Non-PGRN SORT1 Ligands

Several lines of study have emphasized that SORT1 has diverse binding partners, such as pro-forms of NGF and BDNF (Nykjaer et al., 2004; Teng et al., 2005). NGF regulates cell survival and cell death via binding to two different receptors, TrkA and p75NTR (Chao, 2003). In contrast, pro-NGF selectively induces apoptosis through binding to the receptor complex of p75NTR and SORT1. SORT1 is essential for transmitting pro-NGF-dependent death signals via p75NTR (Nykjaer et al., 2004). Similarly, pro-BDNF is an apoptotic ligand that induces death in sympathetic neurons co-expressing SORT1 and p75NTR. The pro-BDNF effect is dependent on cellular co-expression of both p75NTR and SORT1, and competitive antagonists of SORT1 block sympathetic neuron death (Teng et al., 2005). As demonstrated in these reports, the induction of neuron death by pro-NGF or pro-BDNF requires the interaction with SORT1 on the cell surface. Our study showed that anti-SORT1 mAb up-regulated PGRN levels via SORT1 down-regulation. Potentially, such mAbs remove cell surface SORT1 and would prevent the extracellular interaction of SORT1 to pro-NGF or to pro-BDNF which induce neuron death. Therefore, down-regulation of SORT1 by anti-SORT1 mAbs may have various other beneficial effects than up-regulating PGRN. PGRN is known as an anti-inflammatory factor that stimulates regulatory T-cells through TNF-alpha signal activation (Hu et al., 2014), and administration of PGRN reverses inflammatory arthritis in PGRN-deficient mice models of collagen-induced arthritis (Tang et al., 2011). Up-regulation of PGRN by anti-SORT1 mAb may also be applicable to inflammatory diseases such as arthritis. On the other hand, SORT1 is required for BDNF signaling in neuropathic pain (Richner et al., 2019). In a previous report, polyclonal anti-SORT1 antibody prevented neuropathic pain. The authors also indicated that neurotensin (NTS), one of SORT1 ligands (Mazella et al., 1998), could be involved in the prevention of BDNF signaling-mediated neuropathic pain as AF38469, a small-molecule compound of the NTS binding site of SORT1 (Schrøder et al., 2014), inhibited BDNF-induced neuropathic pain. Removal of cell surface SORT1 by anti-SORT1 mAb may block BDNF signaling and anti-SORT1 mAb may be beneficial for the treatment of neuropathic pain, a major clinical challenge resulting from peripheral nerve trauma or disease.

SORT1 also regulates glucose and lipid metabolism. As observed in *Sort1* KO mice (Devader et al., 2016), NTS levels are expected to be up-regulated by anti-SORT1 antibody with SORT1 down-regulation activity. NTS is involved in a wide variety of biological functions, including glucose homeostasis (Blondeau et al., 2019). The NTS-mediated glucose metabolism is likely mediated through SORT1 as evidenced by the fact that *Nts* KO and *Sort1* KO mice show resistance to obesity and hepatic steatosis, and greater insulin sensitivity on a high fat diet as common phenotypes (Rabinowich et al., 2015; Li et al., 2016). These results suggest that anti-SORT1 mAb might be helpful in maintaining glucose homeostasis by up-regulating NTS. SORT1 was shown to act as an uptake receptor for LDL (Strong et al., 2012). They confirmed that plasma LDL is up-regulated in *Sort1* KO mice. While SORT1 also acts as an uptake receptor for VLDL (precursor of LDL) (Sparks et al., 2016), it is expected that SORT1 deficiency induces the up-regulation of extracellular VLDL level. However, when SORT1 is genetically knocked-out in mice, VLDL levels are unchanged or reduced, which is inconsistent with a reciprocal relationship (Sparks et al., 2015). This is likely due to the effect of SORT1 on lipoprotein uptake and export (Strong et al., 2014). Therefore, further studies are required to understand the effect of SORT1 down-regulation by anti-SORT1 mAb on lipid metabolism and homeostasis.

In cells, SORT1 acts as a sorting receptor of multiple proteins, including cathepsin D, cathepsin H, G_M2_AP, prosaposin, and acid sphingomyelinase (Canuel et al., 2009). These proteins use SORT1 to be properly delivered to lysosome and their deficiency causes lysosomal storage disorders. Cathepsin D and acid sphingomyelinase are delivered to lysosomes in both SORT1- and mannose 6-phosphate receptor (MPR)-dependent manner, suggesting that these proteins could be trafficked to lysosomes even when intracellular SORT1 is ablated. However, further research needs to be done to determine if the down-regulation of cell surface SORT1 by anti-SORT1 antibody has an effect on the intracellular sorting function of SORT1 and if lysosomal proteins are properly delivered to lysosomes in the presence of the anti-SORT1 antibody.

## Conclusion

We successfully generated a variety of anti-SORT1 antibodies, and identified those that up-regulated PGRN both *in vitro* and *in vivo*. The primary mechanism of PGRN up-regulation was via enhancing SORT1 down-regulation upon antibody binding. This suggests that SORT1 down-regulation is a key mechanism in increasing PGRN levels by anti-SORT1 antibodies and is a promising target for PGRN boosting therapy in disorders such as FTD-PGRN or arthritis, as indicated by *Pgrn* KO mice phenotype.

## Materials and Methods

### Animal Welfare

All animal-related research protocols used in this study were approved by the Takeda Institutional Animal Care and Use Committee. Animals were handled according to the Guide for the Care and Use of Laboratory Animals (8th edition; National Research Council, 2011).

### Generation of Monoclonal Antibodies

#### Generation of *Sort1*-Knockout (KO) Mice

*Sort1* KO mice were generated as below. Briefly, an approximately 0.5 kbp region including exon3 of the *Sort1* gene was deleted by using target site sequences of 5′-ctgcttcaagtgtaagcgat-3′ and 5′-aagaatccatgagattcgca-3′ in C57BL/6J fertilized eggs by the CRISPR/Cas9 system. Resultant homozygous KO mice were selected by testing the *Sort1* exon3 sequence by qPCR. qPCR primer and probe sequences used were as follows: primers, 5′-TTGTCCCCTGCAGGTTATTCTC-3′ and 5′-ACTGTCCAAAGCTCACAATTACCA-3′; MGB probe, 5′-TCCTGACCACTTTCCAAG-3′.

#### Generation of Anti-SORT1 Monoclonal Antibodies

*Sort1* KO mice (12-weeks old, male and female) were immunized as previously described (Kamala, 2007). Briefly, each mouse was injected subcutaneously in the hock with 5 μg of recombinant human SORT1 protein 5 times twice a week, followed by 5 injections of mouse SORT1 protein. TiterMax Gold (TiterMax) adjuvant was used in the primary immunization and was replaced with the mixture of ODN-1826 (InvivoGen) and aluminium hydroxide (Sigma-Aldrich) for the following boosts. One week after the tenth boost, the final boost was implemented by intraperitoneal injection of 10 μg of mouse SORT1 protein. Three days after the final boost, the lymphocytes from the mice were fused with P3X63Ag8U.1 mouse myeloma cells (ATCC) following standard procedures. Hybridoma selection and cloning were performed using ClonaCel-HY hybridoma kit (STEMCELL Technologies). Culture supernatants were collected from the wells of 96-well plates then screened by automated high-throughput FCM using 300-19 cell lines (purchased from Dr. Naomi Rosenberg’s Lab, Tufts University) expressing human SORT1 or mouse SORT1. Selected hybridomas were cultured in Ham’s F-12 nutrient medium (FUJIFILM Wako Pure Chemical) containing MEM non-essential amino acid solution (FUJIFILM Wako Pure Chemical), sodium pyruvate (FUJIFILM Wako Pure Chemical), L-alanyl-L-glutamine (FUJIFILM Wako Pure Chemical), penicillin and streptomycin (Wako Pure Chemical Industries, Ltd.), and 10% ultra-low IgG fetal bovine serum (Thermo Fisher Scientific) for antibody purification.

#### Preparation of Recombinant Proteins

DNA fragments encoding the extracellular domain of human or murine SORT1 fused with C-terminal 6 × His tag were synthesized and inserted into a pcDNA3.4 vector (Thermo Fischer Scientific). Chimeric proteins were prepared by reference to a previous report (Biilmann et al., 2016). DNA fragments encoding four chimeric proteins of human and puffer fish SORT1 were as below: nABcde, Met1-Arg77 (human)_Arg67-Ser98 (puffer fish)_Gly110-Pro343 (human)_Pro361-Ser773 (puffer fish); NabCDE, Met1-Arg109 (human)_Gly99-Val171 (puffer fish)_Ile202-Pro360 (puffer fish)_Ser344-Asn755 (human); NAbcDE, Met1-K254 (human)_Thr273-Gly538 (puffer fish)_Pro522-Asn755 (human); naBCdE, Met1-R77 (human)_Arg67-Val171 (puffer fish)_I202-Asp272 (puffer fish)_Ala255-Gly521 (human)_ Pro539-Arg623 (puffer fish)_Asp606-Asn755 (human). These chimeric genes were fused to a 6 × His tag-encoding sequence and cloned into a pcDNA3.4 vector. A cDNA fragment encoding human furin M1-A595 C-terminally fused to Flag tag was synthesized and cloned into pcDNA3.4 vector. Recombinant proteins were produced with the Expi293F expression system (Thermo Fisher Scientific) according to manufacturer’s protocol. Expi293F cells were transiently transfected with SORT1-encoding and furin-encoding plasmids and were incubated for 6 days. Culture supernatants were harvested and purified with Ni-NTA excel (GE Healthcare), followed by a SEC column (HiLoad 26/600 Superdex 200) from GE Healthcare.

#### Generation of Stable Cell Lines

300-19 cell lines stably expressing human or mouse SORT1 were generated as previously described (Wang et al., 2018). In order to produce lentiviral particles, HEK293T cells (ATCC) were transiently transfected with pLenti6.2C-V5-DEST vector containing the full-length human mouse SORT1 gene together with Sigma Mission Lentiviral Packaging Mix (Sigma-Aldrich) by using Fugene 6 transfection reagent (Promega) according to the manufacturer’s protocol. Culture medium containing virus was collected 48 h post transfection and precleaned by centrifugation at 2,000 *g* and filtration using a 0.45 μm filter unit (PALL Life Sciences). 300-19 cells were transduced with the viral supernatant and then selected with culture medium containing 1.5 or 9 μg/mL puromycin (Thermo Fisher Scientific) for human or mouse SORT1, respectively. Puromycin-resistant cells were maintained in the puromycin-containing selection medium for 10 days and subcloned by a limiting dilution. Outgrown cells were evaluated by FCM for the expression of human or mouse SORT1.

#### FCM Screening

Hybridoma supernatant samples were screened according to an automated FCM method (Wang et al., 2018). 300-19 cells over-expressing human or mouse SORT1 were labeled with CellTrace Violet (Thermo Fisher Scientific) and Vybrant CFDA SE Cell Tracer (Thermo Fischer Scientific), respectively, following manufacturer’s protocol. The cells were resuspended in cold PBS containing 2% fetal bovine serum (FCM buffer) and incubated with supernatant samples for 30 min at 4°C. After three rounds of washing using cold FCM buffer, 30 μL of Alexa Fluor 647 Anti-Mouse IgG (Jackson ImmunoResearch) was added. After 30 min of incubation at 4°C, the cells were washed twice with FCM buffer, and the binding of antibody was read on an iQue Screener PLUS (IntelliCyt).

### Affinity Measurement of Anti-SORT1 Antibody

An Octet Red96e system (Molecular Devices) based on BLI was used to measure the kinetic parameters of the antibody. First, 10 μg/mL of antibody was captured using AMC Octet biosensors (Molecular Devices) for 120 s. Baseline was determined by an incubation of PBST alone for 60 s. The mAb-capturing biosensors were reacted to recombinant human SORT1 protein at 200 nM to 3.13 nM (R&D Systems) for 120 s followed by dissociation time of 180 s in PBST. The kinetics of the antibody to SORT1 was analyzed with a sensorgram aligned at the beginning of the association step after a background subtraction. The sensorgrams were globally fit to a 1:1 Langmuir binding model.

### Epitope Mapping of Anti-SORT1 Antibody

The epitope mapping study was performed using the Octet Red96e system. Firstly, 10 μg/mL of mAbs were captured using AMC Octet biosensors for 120 s followed by a baseline determination step of 60 s in PBST. The biosensors were reacted to 30 μg/mL of human and chimeric SORT1 proteins for 120 s followed by a dissociation time of 60 s in PBST.

### Epitope Binning of Anti-SORT1 Antibody

The epitope binning of generated anti-SORT1 mAbs was performed by competitive sandwich ELISA. Briefly, the antibodies were immobilized on a 384-well plate (Corning). Separately, biotinylated SORT1 and anti-SORT1 antibody (competitor antibody) were pre-incubated at final concentrations of 100 nM and 1 μg/mL, respectively, and the complex was added to the antibody-coated 384 plate. Plate-bound biotinylated SORT1 was detected using a horseradish peroxidase-conjugated StreptAvidin (Thermo Fisher Scientific) and SureBlue/TMB peroxidase substrate (SeraCare Life Sciences). The reaction was stopped by adding H_2_SO_4_, and theOD_450_ was measured using Wallac ARVO plate reader (PerkinElmer). The binding inhibition (%) was calculated using the following formula:

Bindinginhibition(%)=(1-A/B)×100,

where *A* represents the OD_450_ value of each well and *B* represents the OD_450_ value in a competitor antibody-free well. An epitope clustering was performed with the binding inhibition data by employing Ward’s hierarchical clustering.

### SORT1 Binding ELISA

Binding activities of anti-SORT1 mAbs were tested using ELISA. Briefly, human or mouse SORT1 were immobilized on a 96-well plate and anti-SORT1 mAb was reacted to the plate-bound SORT1. SORT1-reactive mAb was detected using a horseradish peroxidase-conjugated anti-mouse IgG antibody (Jackson ImmunoResearch) and SureBlue/TMB peroxidase substrate. The reaction was stopped by adding H_2_SO_4_, and OD_450_ was measured using a SpectraMax 340PC384 plate reader (Molecular Devices).

### Blocking Ability of Anti-SORT1 Antibody Against PGRN Binding to SORT1

In brief, a human SORT1 expression vector and NeoFection reagent (astec) were mixed at a ratio of 1:1 in OptiMEM I (Thermo Fisher Scientific). After a 15-min incubation, the mixture was added to Expi293 cells at 1 × 10^6^ cells/mL. Two days after the transfection, the cells were mixed with 0.3 μg/mL of biotinylated-PGRN (R&D Systems), 2 μg/mL of StreptAvidin-Alexa Fluor 647 (Thermo Fisher Scientific), and anti-SORT1 antibody in PBS containing 1% fetal bovine serum and 0.05% sodium azide. After a 3-h incubation, the cell surface fluorescence was detected by MirrorBall (TTP Labtech).

### PGRN Clearance Assay With Human U251 Cells

U251 human glioblastoma cells (JCRB) were seeded at a density of 1 × 10^4^ cells/well in a 96-well plate (Corning) in 100 μL of growth media MEM with Glutamax (Thermo Fisher Scientific) supplemented with 10% fetal bovine serum (Invitrogen) and 1% penicillin-streptomycin combination (FUJIFILM Wako Pure Chemical). The cells were incubated at 37°C and 5% CO_2_ for 24 h and treated with the various concentrations of mAbs or PBS and incubated at 37°C and 5% CO_2_ for 72 h. The isotype control antibody used was mouse IgG_1_ (Miltenyi Biotec). Cell supernatant was collected, and ELISA was performed using Human Progranulin DuoSet ELISA (R&D Systems) as per the manufacturer’s instructions. OD_450_ was measured with the ARVO plate reader. Progranulin concentration was normalized against PBS-treated cells to identify relative changes in the progranulin levels.

### Cortical Neuron Culture and PGRN Clearance Assay

Cortical neurons were isolated from E14 C57BL/6 WT mice. In brief, cortices from E14 mice were dissected and dissociated with Neuron Dissociation Solutions S (FUJIFILM Wako Pure Chemical) according to the manufacturer’s instructions. Cells were seeded at a density of 7.5 × 10^4^ cells/well on a Poly-D-Lysine-coated 96-well plate (Corning) and were grown in serum-free Neurobasal Medium (Invitrogen) with B-27 Supplement (Invitrogen), GlutaMAX (Gibco), and 100 U/mL of Penicillin-Streptomycin (Gibco). Half of the media was changed twice weekly. Neurons were used in PGRN clearance assays at 7 DIV. The cells were treated with anti-SORT1 mAb or control mouse IgG_1_ for 7 days. PGRN levels in collected culture media were determined with Mouse Progranulin DuoSet ELISA (R&D Systems) according to the manufacturer’s instructions.

### Detection of SORT1 Down-Regulation

Down-regulation of SORT1 was measured by immunocytochemistry-based image analysis. After the 3-day treatment with anti-SORT1 antibodies (see the section of PGRN clearance assay), the U251 cells were washed with PBS and fixed with 4% paraformaldehyde. The fixed cells were permeabilized with 100 μg/mL of digitonin, incubated with 1 μg/mL of biotinylated anti-SORT1 polyclonal antibody (R&D Systems), and then stained with 2 μg/mL of streptavidin-conjugated Alexa 488 fluorescent dye. The cells were counterstained with 1 μg/mL of Hoechst 33342 (Thermo Fisher Scientific). All reactions were performed in the PBS containing 0.1% bovine serum albumin. Microscopic fluorescent images were obtained by In Cell Analyzer 6000 (GE Healthcare), followed by the image analysis using the In-Cell Developer program (GE Healthcare). The number of nuclei was used for normalization.

### Plasma PGRN Measurement

Three to four C57BL/6J mice (8 or 9-weeks old, male; CLEA Japan) were intravenously injected with anti-SORT1 antibody, clone K1-67, at 100 mg/kg. After 1 or 3 days, the mice were anesthetized with isoflurane to collect CSF and blood from the abdominal aorta by using heparin as an anticoagulant. The blood samples were centrifuged at 12,000 rpm for 10 min at 4°C for plasma isolation. Because of the small volumes obtained from each animal, CSF from 4 mice was pooled for PGRN measurement. PGRN levels were analyzed using a Mouse Progranulin Quantikine ELISA Kit (R&D Systems) according to the manufacturer’s protocol. Statistical analysis was performed (K1-67-treated versus PBS-treated groups) using the Aspin–Welch *t*-test.

### Plasma K1-67 Measurement

Plasma K1-67 levels were analyzed by immunocapture-liquid chromatography-mass spectrometry according to the procedure described in the literature (Hashii et al., 2018). Briefly, plasma K1-67 was immunocaptured by Dynabeads Protein G (Thermo Fisher Scientific) with a KingFisher Flex magnetic particle processor (Thermo Fisher Scientific), and then digested by trypsin (Promega). The digested samples were purified with Oasis MCX μElution plate (Waters) and then subjected to liquid chromatography-mass spectrometry. The peptide sequence of K1-67 (TAQATAYWGQGTLVTVSAAK) was specifically detected in the plasma and monitored by selected reaction monitoring analysis under the positive ion mode with a mass transition of m/z 1012.5 to 526.3 (precursor ion to product ion). Statistical analysis was performed (K1-67-treated versus PBS-treated groups) using the Aspin–Welch *t*-test.

### Microdialysis

Mice were anesthetized using isoflurane. The skin over the skull was cut and separated from the skull surface. With bupivacaine topically applied to the skull, three small holes were made in the bone of the skull at the target site. Guide cannulas (CMA microdialysis) were placed into the holes. Sterile obturators were inserted into each guide cannula to prevent infections or the formation of obstructions and remained in place except during testing. Animals were allowed to recover for 7 days after surgery. Following the procedure, at least one post-operative dose of Rimydal 5 mg/kg and a second dose 24 h later were administered. Twenty-four hours before the start of the microdialysis experiment, the animals were treated with PBS, K1-67 at 50 mg/kg or 100 mg/kg. On the day of the experiment, a 1,000 kDa cut-off probe (CMA Microdialysis) was inserted via the guide cannula into the hippocampus. The probe was connected to a microdialysis peristaltic pump (Microbiotech). The inlet tubing of the microdialysis probe was connected to a peristaltic pump perfusing the probe with artificial CSF. The peristaltic pump was also connected to the outlet tubing in order to prevent perfusion fluid loss from the probe, by pulling the fluid through the tubing. A perfusion buffer, 25% bovine albumin fraction V (Sigma-Aldrich), was diluted to 0.2% with artificial CSF (147 mM NaCl, 2.7 mM KCl, 1.2 mM CaCl_2_, 0.85 mM MgCl_2_) on the day of use. The pump was set to a constant flow of 1 μL/min. A 2-h sampling regimen was used throughout the experiment providing 12 samples over a 24 h collection period. Statistical analysis was performed (K1-67-treated versus PBS-treated groups) using the two-way RM ANOVA analysis with Sidak’s multiple comparisons.

## Data Availability Statement

The datasets generated for the figures and table shown in this study are available upon request to the corresponding author.

## Ethics Statement

The animal study was reviewed and approved by the Takeda Institutional Animal Care and Use Committee.

## Author Contributions

SM profiled anti-SORT1 antibodies, performed epitope binning, affinity measurement, PGRN-SORT1 interaction, and epitope domain identification, and wrote the manuscript. HSa executed SORT1 down-regulation assay and wrote the manuscript. DW performed U251 PGRN up-regulation assay and wrote the manuscript. SA carried out mouse primary neuron PGRN up-regulation assay and wrote the manuscript. NA conducted *in vivo* PGRN up-regulation study and wrote the manuscript. TY generated anti-SORT1 antibodies and wrote the manuscript. DT supervised anti-SORT1 antibody discovery and wrote the manuscript. AH performed anti-SORT1 antibody screening and wrote the manuscript. KId prepared chimeric SORT1 proteins and wrote the manuscript. YH conducted *in vivo* PGRN up-regulation study. YO analyzed epitope binning data. SY measured SORT1 antibody in mouse blood and wrote the manuscript. KIi and HSh measured SORT1 antibody in mouse blood. SK supervised anti-SORT1 antibody study. SS critically reviewed and revised the manuscript. All authors contributed to experiment designs and coordinated the studies. All authors have read and approved the final version of the manuscript.

## Conflict of Interest

The authors of the publication were employees of the Takeda Pharmaceutical Company at the time the research was conducted.

## Abbreviations

AMC: anti-mouse Fc
BBB: blood-brain barrier
BLI: biolayer interferometry
CSF: cerebrospinal fluid
DIV: days *in vitro*
ELISA: enzyme-linked immunosorbent assay
FCM: flow cytometry
FcRn: neonatal Fc receptor
FTD: frontotemporal dementia
ISF: interstitial fluid
KO: knockout
mAb: monoclonal antibody
LDL: low-density lipoprotein
NTS: neurotensin
OD_450_: optical density at 450 nm
PGRN: progranulin
PBS: phosphate-buffered saline
PBST: PBS containing 0.05% tween-20
SD: standard deviation
SORT1: sortilin1
VLDL: very low-density lipoprotein
WT: wild-type.
